# Cholera outbreak among the Sama Badjao Indigenous community, Lucena City, Philippines, 2022

**DOI:** 10.5365/wpsar.2026.17.2.1223

**Published:** 2026-06-30

**Authors:** Iris Ann D Bigyan, Catherine P Patricio, Johnette A Peñas, Nelson C Soriano, John Bobbie M Roca, Ray Justin Ventura, Mariz Zheila Blanco-Payuyo, Apple Charm Agulto

**Affiliations:** a Quezon Provincial Health Office, Quezon, Philippines.; b Lucena City Health Office, Quezon, Philippines.; c Local Government Unit, San Jose de Buan, Samar, Philippines.; d Cavite Provincial Health Office, Cavite, Philippines.; e Center for Health Development, Regional Office IV-A, Department of Health, Manila, Philippines.; f Department of Health, Manila, Philippines.; g Field Epidemiology Training Program Alumni Foundation, Inc., Manila, Philippines.

## Abstract

**Objective:**

On 18 October 2022, diarrhoeal cases among the Sama Badjao indigenous community in the Philippines were reported from a tertiary hospital in Lucena City, Quezon Province. An investigation was conducted to confirm the outbreak, profile cases, identify the source of infection, and recommend prevention and control measures.

**Methods:**

Suspected cases were identified through medical records review, active case finding and key informant interviews. Identified cases were interviewed using a structured questionnaire to collect demographic, clinical and exposure information. Rectal swabs and environmental water samples were collected for bacterial isolation and confirmation.

**Results:**

A total of 75 cases were identified, with 40 (53.3%) hospitalized and four fatalities (case fatality rate: 5.3%). Ages ranged from 2 months to 20 years (median: 4 years). Nine of the 15 rectal swabs were positive for *Vibrio cholerae* Ogawa biotype El Tor. The community practised open defecation, and only 24.7% of households had basic sanitation. Although *V. cholerae* was not found in water samples, faecal contamination was noted in a community well, and *Aeromonas* species were isolated, indicating the presence of multiple gastrointestinal pathogens.

**Discussion:**

A cholera outbreak was confirmed among the Sama Badjao community in Lucena City. Environmental assessment indicated that poor sanitation, open defecation and unsafe water sources likely contributed to the outbreak. The high case fatality rate (5.3%) was influenced by delayed care and limited health-care access. These findings emphasize the need for interventions to improve water, sanitation and hygiene practices and provide culturally appropriate public health programmes to reduce cholera risk among vulnerable communities.

Cholera, an acute bacterial infection caused by *Vibrio cholerae*, is primarily transmitted through contaminated food or water, leading to severe watery diarrhoea that can cause dehydration and death if untreated. The disease is a significant public health issue in regions with inadequate access to safe water, sanitation and hygiene. (*1*)

In the Philippines, cholera is endemic, with cases regularly reported from several regions. However, underreporting of cases is common due to variations in local surveillance capacity and laboratory availability, complicating the understanding of its epidemiology. Few analyses of surveillance data are available, and detailed outbreak investigations are limited. (*2*) Nonetheless, past outbreaks, such as one in Nabua, Camarines Sur in 2012, which resulted in 309 suspected cases and two fatalities, demonstrate the critical influence of water and sanitation conditions on cholera transmission. The outbreak was linked to unchlorinated water sources and inadequate sanitation, while access to piped water proved protective against the disease. (*3*)

Lucena City, the capital of Quezon Province, is a highly urbanized coastal city with a population of 278 924 (2020 census). One of the city’s 33 barangays (administrative units), Dalahican, has 5563 households and a population of 24 640, of whom around 10% (*n* = 2465) are members of the Sama Badjao community. The Sama Badjao are an Indigenous Philippine group who live in stilt houses above shallow waters and practise herbal medicine. They face barriers to health-care services – such as distance, language and perceptions of quality – that lead to reliance on traditional healing and poorer health outcomes. (*4*)

On 18 October 2022, a nurse in Lucena City reported an increase in acute gastroenteritis admissions, including two deaths, from the Sama Badjao community. The Quezon Provincial Epidemiology and Surveillance Unit confirmed this incident as a potential outbreak. A team from the Field Epidemiology Training Program conducted an investigation on 19 October to confirm the outbreak, profile cases and deaths, identify the infection source, and recommend prevention and control measures.

## Methods

### Descriptive study

Medical records from health facilities most likely to have received patients from Dalahican were reviewed, including the Barangay Health Station and referral hospitals in Lucena City. The review focused on patients with suspected cholera or acute gastroenteritis, particularly watery diarrhoea, to create a case list. Demographic data included age, sex, address, consultation/admission date and symptoms. Records from the Philippine Integrated Disease Surveillance and Response system for Lucena City from the previous 5 years (2017–2021) were also analysed. Active case finding involved house-to-house visits within the Sama Badjao community, during which cases were interviewed using a standard questionnaire (**Supplementary Material**). A suspected case was defined as any previously well resident of the Sama Badjao community experiencing acute watery diarrhoea (three or more episodes per day) from 1 October to 2 November 2022, while a confirmed case was a suspected case who tested positive for *V. cholerae*.



### Key informant interviews

The barangay captain was interviewed to understand the lifestyle and health-related attitudes and behaviours of the Sama Badjao community. Hospital staff were also interviewed about the diagnosis and management of diarrhoeal cases.

### Clinical and environmental sampling

Rectal swab samples were collected from 15 individuals with active watery diarrhoea meeting the criteria for suspected cases. These samples were placed in Cary-Blair transport medium and transported to the Research Institute for Tropical Medicine (RITM) for bacteriological analysis.

Concurrently, an environmental investigation was conducted to assess water sources, waste management practices, availability of sanitary toilets, and overall water and sanitation conditions in the community. Interviews with local leaders and residents revealed that a communal dug well and a water refilling station were the primary sources of drinking-water. Ten water samples were collected: two from each of five sites – two household water containers, the dug well, a water tank at the refilling station, and raw water from the refilling station. Samples were collected aseptically in sterile 500 mL bottles; one set of samples from each site was sent to RITM for bacteriological analysis, while the second set was sent to the Provincial Water Laboratory to test for total coliforms and *Escherichia coli* using Colilert-18. Additionally, operational records from the water refilling station were reviewed.

## Results

### Case characteristics

**Fig. 1:**
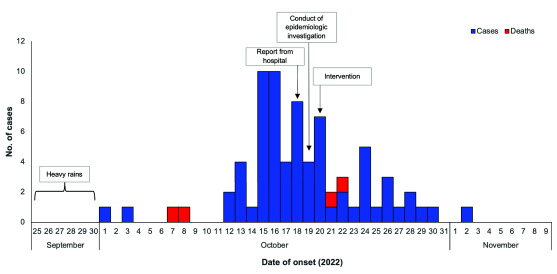
Cholera cases by date of onset of illness, Sama Badjao community, Dalahican, Lucena City, Philippines, 1 October-2 November 2022 (*N* = 75)

A total of 75 suspected cholera cases were identified through active case finding and health facility records review. The earliest reported onset of illness was on 1 October 2022, with a peak in cases occurring on 15 and 16 October 2022 (**Fig. 1**). Four deaths were recorded, all in children, for a case fatality rate (CFR) of 5.3%.

[**insert** Figure 1]

Cases ranged in age from 2 months to 20 years, with a median of 4 years. The most affected age group was 0–4 years, and 39 (52.0%) were male. All cases had acute diarrhoea; 31 (41.3%) had vomiting, 21 (28.0%) had abdominal pain, and 16 (21.3%) had fever. Hospitalization was necessary for 40 (53.3%) cases (**Table 1**). Interview responses revealed that 60 (80.0%) of the cases did not wash their hands before eating, and 70 (93.3%) did not wash their hands after defecation.

**Table 1:** Characteristics of suspected cholera cases among the Sama Badjao community, Dalahican, Lucena City, Philippines, 1 October–2 November 2022 (*n* = 75)

Characteristics	No. (%) of cases
Sex	
Male	**39 (52.0)**
Female	**36 (48.0)**
Age group, years	
0–4	**39 (52.0)**
5–9	**20 (26.7)**
10–14	**11 (14.7)**
15–19	**4 (5.3)**
≥ 20	**1 (1.3)**
Signs and symptoms^a^	
Acute watery diarrhoea	**75 (100)**
Vomiting	**31 (41.3)**
Abdominal pain	**21 (28.0)**
Fever	**16 (21.3)**
Body weakness	**11 (14.7)**
Sunken eyeballs	**10 (13.3)**
Hospitalized	
Yes	**40 (53.3)**
No	**35 (46.7)**

^a^ Multiple responses were allowed.

### Key informant interviews

The barangay captain indicated that no social gatherings took place in the weeks leading up to the incident, although children began experiencing diarrhoea following heavy rains at the end of September (**Fig. 1**). Additionally, the captain noted that there had been no severe diarrhoeal cases in the village over the past 5 years. A review of surveillance records confirmed the absence of reported cholera cases and outbreaks of acute watery diarrhoea during the same period.

A health worker from the Sama Badjao community indicated that the local population lacks awareness regarding the risks associated with acute diarrhoea, only seeking medical assistance after the fatalities of two children early in the outbreak (**Fig. 1**). The health worker noted that Sama Badjao individuals generally visit health stations primarily to acquire medicines, rather than for medical consultations.

### Environmental investigation

The communal dug well was shallow, unprotected, and located 50 m from homes, where it was surrounded by debris and lacked a waste disposal area. Residents reported that the water frequently turned brown during heavy rains, and it was rarely treated before use.

The houses, mainly built from light materials, often had makeshift sanitary toilets, with elderly individuals using recycled pails for this purpose. Faecal waste was disposed of underneath houses into the water below, which also doubled as bathing areas.

### Laboratory results

Of the 15 rectal swab samples taken, nine (60.0%) were positive for *V. cholerae* Ogawa biotype El Tor. *Aeromonas* (*hydrophilia* and *veronii* biovar) were also isolated in two (13.3%) samples.

All five water samples tested for chromogenic substrate (Colilert-18) at the Provincial Water Laboratory contained total coliforms and *E. coli*. Two of the five samples subjected to bacteriological analysis at RITM – the dug well and the raw water from the refilling station – tested positive for *Aeromonas caviae* and *A. veronii* biovar *sobria*; none tested positive for *V. cholerae.* The records review revealed that water testing at this refilling station was conducted regularly and that water samples passed quality checks, except during a period in September 2022.

### Follow-up action: prevention and control measures

After the investigation, local health officials conducted household education, emphasizing household chlorination, boiling water, good hygiene practices and protecting water sources from contamination. They advised sick individuals to avoid participating in food preparation and provided clean water and rehydration sachets. Barangay health workers received refresher training on diarrhoea case notification, emphasizing the need for timely reporting, and reminded community members to seek health care promptly if symptoms appear. A water quality monitoring committee was formed to ensure safe water access and improve coordination among agencies and the Sama Badjao community for sustainable water, sanitation and hygiene practices.

## Discussion

The investigation confirmed an outbreak of cholera among the Sama Badjao community in Dalahican, Lucena City. Environmental findings suggested that contaminated water sources may have contributed to transmission. Although *V. cholerae* was not detected in the water samples, several factors point to the open dug well as the most probable source of transmission. The shallow well was unprotected and located close to households, and testing showed faecal contamination, indicating poor water quality. The outbreak followed heavy rainfall that may have introduced contamination via runoff, a risk exacerbated by the community’s practice of open defecation.

Raw water from the refilling station was also considered a potential source of exposure. *Aeromonas* species (*A. caviae* and *A. veronii* biovar) were isolated from both the open dug well and the refilling station’s raw water, indicating faecal contamination. Two suspected cases tested positive for *Aeromonas*, which are recognized as gastrointestinal pathogens that produce symptoms similar to cholera. (*5*, *6*) The detection of *Aeromonas* in both water sources suggests that residents may have been exposed to multiple enteric pathogens through contaminated water.

A lack of access to sanitation is recognized as a major contributor to the magnitude and severity of cholera outbreaks. A 2021 report from the Electronic Field Health Information System of Lucena City indicated that only 1363 (24.7%) households in Dalahican had access to basic sanitation facilities (report available upon request). The Sama Badjao settlement exemplified severely unsanitary conditions with a lack of toilets, widespread open defecation and reliance on untreated water sources, creating a favourable environment for waterborne diseases such as cholera. Similar risk factors have been documented in cholera outbreaks in other vulnerable communities, (*7*-*10*) such as during a cholera outbreak in Somalia, where inadequate sanitation, unsafe drinking-water, poor hygiene practices and limited awareness of cholera exacerbated disease transmission. (*8*) While both regions are geographically distinct, they share common structural challenges, including poverty, insufficient water and sanitation infrastructure, and limited access to health services.

Laboratory investigations indicated that the cholera outbreak was caused by *V. cholerae* Ogawa biotype El Tor. This strain is notable for its ability to persist in environmental water sources, (*1*) effective host-to-host transmission and a higher occurrence of asymptomatic carriers. (*11*) While El Tor strains typically lead to milder disease compared to classical strains, (*12*) the outbreak nonetheless had a CFR of 5.3%, which surpasses the target of below 1% for cholera outbreaks. (*13*) Delayed medical care likely contributed to this elevated CFR, as some community members only sought assistance after the deaths of two children.

The vulnerability of the Sama Badjao community to cholera highlights broader social health inequities. Traditionally nomadic, these communities face difficulties adapting to urban environments. Research in other areas where the Sama Badjao reside, such as Bongao, Tawi-Tawi Province, has documented persistent poverty and limited access to essential services such as education and health care, (*4*) contributing to their increased risk of infectious disease outbreaks.

Preventing future outbreaks in similar settings requires an integrated approach that combines improvements in water and sanitation infrastructure with culturally sensitive community engagement. Key measures include increasing access to safe drinking-water, improving sanitation facilities and promoting good hygiene practices. (*14*) Community health education and improved access to early treatment are also vital for reducing morbidity and mortality during cholera outbreaks. Additionally, strengthening public health policies that prioritize Indigenous Peoples and other vulnerable populations can help address health inequities. (*15*)

This study had several limitations. First, the source of the outbreak was undetermined, with environmental findings suggesting contaminated water, particularly from the open dug well, though all water samples tested negative for *V. cholerae*. This ambiguity could be due to the timing of sample collection, dilution of contaminants during heavy rainfall or limitations of the sampling method. Second, case numbers may be underestimated, as mild cases might not have sought medical care. Laboratory confirmations were limited by a shortage of testing kits, thereby reducing the detection of additional cases and other pathogens. Finally, while *Aeromonas* species were detected in samples, the available diagnostic methods could not adequately distinguish their role in the outbreak relative to *V. cholerae*. Despite these challenges, the study offered valuable insights into the outbreak's environmental and social factors, guiding public health responses in a vulnerable Indigenous community.
