# Supplementary material for: Cholera outbreak among the Sama Badjao Indigenous community, Lucena City, Philippines, 2022
Source: Western Pac Surveill Response J. 2026 Jun 30;17(2):1–6. doi: 10.5365/wpsar.2026.17.2.1223 (PMC13389470; doi:10.5365/wpsar.2026.17.2.1223)
Supplement: Supplementary file 1 [file wpsar-17-1223-s001.pdf]

Supplementary Material. **Questionnaire for suspected cholera cases in Dalahican Village, Lucena City, Philippines, 2022**

QUESTIONNAIRE FOR DIARRHOEA CASES

Name: ______________________ Sex: ________ Age: ________

Address: ______________________

House No. ________ Purok Sitio Barangay

Occupation: ______________________ Place of work/school ______________________

Clinical information Signs & symptoms (pls. check)

Onset time		Loose bowel movement >3x/day	Yellowish
Onset date		Fever	Greenish
Illness duration		Nausea	Others: specify
Diagnosis date		Vomiting	
Hospitalized (Y/N)		Body weakness	
Date of admission		Abdominal cramps	
Date of discharge		Rectal pain	Volume of stool/episode
Name of hospital		Tenesmus	Scanty
Outcome		Bloody stool	Voluminous
		Brownish	Quantify:

Other household members affected: (name, age, sex, and onset date)

1. ______________________
2. ______________________
3. ______________________

Water and Sanitation

1. Before the LBM arises, do you boil your water? Yes ( ) No ( )
2. Do you use chlorine in your drinking-water? Yes ( ) No ( )
3. Do you wash your hands before eating? Yes ( ) No ( )
4. Do you wash your hands after using the toilet? Yes ( ) No ( )
5. Do you have your own toilet? Yes ( ) No ( ); water sealed, pit privy, others: ________
6. Do you use ice? If yes, homemade, bought? Where? ________
7. Source of drinking-water? Water system, deep well, spring bottled, refilling station, rain water, specify where? ________
8. Approximately how many glasses of water do you consume per day? ________
9. Did you attend a party prior to the occurence of diarrhoea? Yes ( ) No ( )
10. Marketplace you preferred: ________
11. Are you a watcher of patient that was confined in the hospital due to diarrhoea? Yes ( ) No ( )
12. If yes, did you develop diarrhea? Yes ( ) No ( )
